# Evaluation and Implementation of the Gamified Jooay Mobile Health App: Protocol for a Hybrid Implementation-Effectiveness Study

**DOI:** 10.2196/97008

**Published:** 2026-09-04

**Authors:** Ebrahim Mahmoudi, Gabriela Szydlowski, Mehrnoosh Movahed, Annette Majnemer, Keiko Shikako

**Affiliations:** 1 School of Physical and Occupational Therapy, Faculty of Medicine and Health Sciences, McGill University, 3654 Prom Sir-William-Osler, Montréal, QC, H3G 1A9, Canada, 1 514 488 5552; 2 Research Institute of the McGill University Health Centre, Montreal, QC, Canada; 3 Centre for Interdisciplinary Research in Rehabilitation, Montreal, QC, Canada; 4 Ottawa Hospital, Ottawa, ON, Canada

**Keywords:** gamification, mobile health, knowledge brokers, implementation science, effectiveness-implementation, Consolidated Framework for Implementation Research, CFIR, capability, opportunity, motivation–behavior, COM-B

## Abstract

**Background:**

Leisure participation is essential for children’s health and well-being and is also a fundamental human right; however, children and youth with disabilities face significant barriers to participation compared with their peers without disabilities. The Jooay App was developed to address gaps in information and access to adaptive and inclusive leisure activities. A new gamification-based version of the Jooay App was co-designed with users, including youth with disabilities, parents, and health care professionals.

**Objective:**

This study aims to describe a study protocol to implement and evaluate the gamified version of the Jooay App using a patient-oriented approach to increase awareness about adaptive and inclusive leisure activities and promote participation in leisure among children and youth with disabilities.

**Methods:**

A hybrid type II effectiveness-implementation study will be conducted. The Consolidated Framework for Implementation Research (CFIR) will guide implementation strategies, including the expansion of knowledge broker (KB) networks to raise the profile of adaptive and inclusive leisure activities in Canada. An interrupted time series (ITS) design will be used to evaluate implementation outcomes across 5 provinces in Canada. Process evaluation will analyze inner and outer setting characteristics, KB strategies, and community resource mobilization. Effectiveness outcomes will be assessed through two phases: (1) a nonrandomized mixed methods pilot feasibility study of the app, where acceptability, engagement, implementation, and potential benefits will be measured, and (2) an ITS at the population level, where overall app usage (eg, clicks, logins, and time spent), individual gamification feature metrics, and user feedback will assess effectiveness outcomes.

**Results:**

The project was funded by the CHILD-BRIGHT (Child Health Initiatives Limiting Disability–Brain Research Improving Growth and Health Trajectories) Network through the Strategy for Patient-Oriented Research, Canadian Institutes of Health Research, in September 2025, and data collection is expected to occur from March 2026 to June 2027. As of manuscript submission, 12 KBs have been recruited, the ITS study and data analysis have not yet begun, and the study results for the implementation and effectiveness components are expected to be published in fall 2027. Findings will be disseminated via peer-reviewed publications, conference presentations, educational materials, social media, community events, and reports. Outputs will include a summary of findings in lay language, in both French and English, including short videos, infographics, and posters for various social media platforms. Community organizations will assist in further dissemination through their networks.

**Conclusions:**

This study will evaluate both the effectiveness and the implementation of the gamified Jooay mobile app to promote participation in leisure among children and youth with disabilities using a hybrid effectiveness–implementation design. By applying the CFIR framework, this study aims to generate evidence about how the Jooay App can be effectively implemented across Canada and expand scholarship on the implementation of technology for equity-deserving groups.

## Introduction

### Background and Rationale

Participation is an essential component of childhood development and is a human right for children and youth [1,2]. However, children and youth with disabilities experience limitations in participation in leisure activities compared to those without disabilities [3]. Their limited participation can be attributed to various barriers, including physical (eg, accessibility and built environment), social (eg, peer support), attitudinal (eg, perceptions of disability), and institutional factors (eg, policies and the availability of adapted programs) [4]. Additionally, a critical barrier that requires attention is the lack of accessible information regarding adaptive and inclusive leisure activities, which further hinders participation [5].

Developed in 2015, the Jooay App is a mobile health (mHealth) solution designed to address this community need. Since its launch, its user base has steadily grown, with reported benefits including integration into rehabilitation and education interventions, physician-endorsed health promotion, increased awareness among parents and youth, and greater visibility for community leisure programs [6].

Preliminary studies indicate that information on adaptive and inclusive leisure programs is unevenly distributed across Canada, with lower availability in provinces experiencing greater social and material deprivation and outside urban centers [7]. Provincial dialogs with interest holders and local knowledge broker (KB) networks have been identified as effective in raising awareness and expanding knowledge of these activities [8]. Additionally, various strategies, such as in-app surveys and tailored responses during the COVID-19 pandemic, have been tested to enhance user engagement [9]. Our latest study suggests that incorporating targeted engagement strategies within the app, in addition to a targeted implementation strategy with various KBs, could further promote participation in leisure activities [6].

Gamification, which is integrating game-like features into mobile apps, can enhance user engagement and can promote health behavior change in various populations [10]. The use of mHealth and gamification in health care is expanding, with studies exploring its potential for encouraging healthy behaviors in children with disabilities. However, such technologies often lack user-centered design [11]. To address this gap, we co-designed a gamified version of the Jooay App following human-centered co-design principles, incorporating insights from youth with disabilities, parents, clinicians, and community organizations through interviews, co-design sessions, and usability studies [12].

mHealth interventions can enhance access to evidence-based services. However, achieving successful implementation and ensuring their long-term sustainability in real-world contexts remain complex challenges. Factors such as poor integration within existing systems, limited involvement of key interested parties, and the lack of sustained support mechanisms often result in low uptake and inconsistent use, even when the intervention itself is well-designed [13]. Increasingly, it is recognized that effective implementation requires tailored, theory-driven, and contextually responsive strategies that address barriers at the organizational, provider, and patient levels, rather than relying solely on passive dissemination or technological innovation [14].

Community-based interventions, particularly those involving mHealth tools like the gamified Jooay App and KB networks, face distinct implementation and sustainability challenges stemming from their reliance on multilevel coordination across sectors (eg, health, education, recreation, and municipal services), voluntary engagement, and context-specific adaptation [15]. Unlike initiatives embedded within a single organization, these interventions depend on partnerships with diverse interest holders operating under varying mandates, resources, and priorities, leading to variability in local infrastructure, leadership support, and organizational readiness that can affect uptake, fidelity, and equitable reach across settings—especially in rural or socioeconomically disadvantaged provinces of Canada, where information access remains uneven [16]. Key barriers include logistical difficulties in recruiting and retaining diverse KBs from backgrounds such as health care providers, educators, families, and youth with disabilities, which may compromise representation and contextual relevance [17]. Sustained engagement of community partners is often constrained by limited funding, staff turnover, competing priorities, resource inequities, and inadequate ongoing support mechanisms post initial implementation [15]. Broader sustainability issues for such multisystem efforts encompass challenges in aligning the app and KB strategies with existing health care, educational, and community systems without continuous external resources, often resulting in uneven dissemination, inconsistent mobilization of local networks, reduced uptake, and eventual fade-out, even with strong initial design and theory-driven frameworks like the Consolidated Framework for Implementation Research (CFIR).

KB networks are known as effective ways to implement public health interventions [8], especially for complex interventions that are not localized or delivered directly by a health care provider and require multiple systems and communities to interact for their collective benefit [18]. The KB is an individual who represents an interest group or an organization representative who “synthesizes local community and patient data with general and specific research knowledge to assist users in translating the evidence into locally relevant recommendations for policy and practice” [19]. The KB’s knowledge of the local context (eg, provincial resources and policies supporting leisure, or local school board or municipality leisure incentives) can help leverage the community resources and ensure the best dissemination strategies are used.

### Objectives

The primary objective of this study is to evaluate whether the deployment of the gamified version of the Jooay App is associated with changes in app engagement compared with the previous nongamified version. Specifically, we will examine changes in app engagement following the deployment of the gamified Jooay App, using an interrupted time series (ITS) analysis of aggregated app analytics data.

The secondary effectiveness and feasibility objectives are to examine changes in specific engagement indicators, including app sessions, active users, duration of use, frequency of use, and use of key app features; to evaluate the feasibility of the gamified Jooay App among youth with disabilities, parents, and clinicians; and to explore postdeployment participation-related outcomes, including planned activities, attended activities, satisfaction, and perceived achievement.

The implementation objectives are to assess the process of implementing and scaling up the gamified Jooay App across participating Canadian provinces; to describe the expansion, role, and activities of the Jooay KB networks across these provinces; to identify provincial barriers and facilitators influencing app uptake, implementation, and sustainability; and to examine how the app and KB networks support the identification of community-based play and leisure opportunities for children and youth with disabilities.

## Methods

### Study Design

We will adopt a hybrid type II effectiveness-implementation design, testing the implementation strategy while observing and gathering information on the intervention’s impact on relevant outcomes [20,21].

### Theoretical Framework

In this study, we will use the CFIR to implement and evaluate the scale-up and expansion of the app use through the expansion of KB networks [22]. KB networks will mobilize information about the app and foster engagement with the app and community activities. We will use the health behavior framework to guide the individual intervention evaluation for the user-designed gamification features to increase individual engagement with the app [23].

### Sampling

#### Preimplementation

The preimplementation phase involves a human-centered co-design process to identify and integrate evidence-based gamification features into the Jooay App. Additionally, we will codevelop informational materials to guide KBs and users in maximizing app engagement. KBs will receive training on the gamified app, along with codeveloped dissemination materials (eg, videos and infographics). Training will emphasize active engagement, including writing reviews, adding activities, notifying organizations of their inclusion in the Jooay database, participating in the “Community Connecting to Play” shared KB journals, and engaging with Jooay social media.

#### Implementation

The KB strategy will be implemented and evaluated using an ITS. This approach is well-suited for assessing the effectiveness of population-level health interventions and testing their impact on specific outcomes within a defined impact model. We will conduct a multisite ITS study, accounting for the KB interventions in 5 different provinces. KBs who participated in previous Jooay studies will be invited to participate in a focus group to (1) review past KB practices, (2) rank strategies based on perceived effectiveness, (3) identify contextual factors influencing implementation, and (4) refine strategies for different settings. New KBs will be recruited and trained through interactive online workshops in purposefully selected provinces (British Columbia, Alberta, Saskatchewan, Ontario, Québec, and Yukon) and across sectors (families, youth, health, and education). KBs will identify new activities, update program information in their provinces, identify populations who may not know about the app, and develop strategies to disseminate within these provinces and promote the app to users (youth, families, clinicians, and educators) and community organizations within their scope of reach. Their insights will inform a 2-fold approach to innovation, sustainability, and use: (1) an automated system to optimize resource identification and updates within the Jooay database, and (2) an organic, human, and community-led dissemination process.

#### Effectiveness

We will evaluate the effectiveness of the app use on users’ engagement and participation in the community through two phases: (1) a nonrandomized mixed methods pilot feasibility study at the individual level and (2) an ITS study of the Jooay App implementation at the community level. Participants will include clinicians, parents of youth with disabilities of all ages, youth with disabilities aged 14 years and older, and educators. They will be recruited from the current user-base of the Jooay App (over 6000 users across Canada), through rehabilitation centers, community organizations, and schools that are part of the Jooay App research group, and social media and partner organizations’ mailing lists.

### Sample Size

This study includes three different sample-size considerations: (1) the ITS analysis of app engagement outcomes at the population level; (2) the recruitment of KBs to support the implementation of the app across Canadian provinces; and (3) qualitative interviews with KBs and feasibility study participants. Because these research components have different research objectives, the sample-size rationale differs across study components, as described below. For the ITS analysis, the primary unit of analysis will be the repeated time interval. ITS emphasizes the importance of repeated observations, underlying trends, and seasonal patterns when evaluating population-level interventions introduced at a clearly defined time point [24]. Therefore, engagement outcomes will be extracted from in-app analytics and aggregated monthly at the community level across all eligible app users. We will include a minimum of 12 monthly time points before and 12 monthly time points after the deployment of the gamified version of the Jooay App.

KBs will serve as provincial implementation partners who support raising awareness about the app across Canada. We will recruit at least 1 KB from each target province, for a minimum of 6 KBs, with the possibility of recruiting up to 2 per province where feasible. This will be determined during the preimplementation phase with current KBs and patient and community partners of the Jooay App research team. This approach will help us ensure sufficient representation across Canadian provinces, Jooay user types (eg, clinicians and parents), and communities with varying availability of adaptive and inclusive leisure activities. Qualitative interviews do not preclude a sample size. Purposeful, maximum variation sampling will be adopted to recruit KBs and feasibility study participants across sectors and regions of interest, with varied experiences and levels of engagement with leisure and their communities. We will also aim to recruit participants in regions where we know there is a limited offer of community-based activities to develop strategies that can support increased participation at the individual and community levels.

### Setting

#### Implementation

This study will be conducted in 5 provinces and 1 territory in Canada, with a focus on health care and community settings. A purposeful sampling strategy and a maximum variation sampling technique will be used to recruit KBs across provinces (British Columbia, Alberta, Saskatchewan, Ontario, Québec, and Yukon—these are the provinces where Jooay activities are most developed and previous KB strategies have been used), living in urban, suburban, and rural areas, and speaking French or English.

#### Effectiveness

For the pilot feasibility study of the mobile app, youth with different disabilities from different provinces and age groups (14‐29 y), parents, clinicians, and educators, living in urban, suburban, and rural areas, and speaking French or English will be recruited.

### Procedures

#### Implementation

KBs will be recruited across multiple Canadian provinces (British Columbia, Alberta, Saskatchewan, Ontario, Québec, and Yukon) and sectors (health, education, community organizations, families, and youth networks). Recruitment will follow a purposive and network-based strategy. Existing KBs and community partners involved in previous Jooay initiatives will first be invited to participate in an online focus group to review prior brokering strategies and identify contextually relevant approaches for scale-up. Additional KBs will be identified through professional networks, stakeholder recommendations, and community organizations (“natural” KB networks), including clinicians, educators, rehabilitation program managers, community leaders, and information hubs. Interested individuals will receive an email invitation outlining study objectives, procedures, and time commitment. Electronic informed consent will be obtained prior to participation. KBs will receive CAD $100 (CAD $1=US $0.72 as of March 1, 2026) per month for 6 months in recognition of their participation.

Prior to implementation, KBs will complete an online readiness-to-implement questionnaire informed by the capability, opportunity, motivation–behavior (COM-B) model [25]. The questionnaire will assess capability (knowledge of Jooay and inclusive leisure resources), opportunity (access to relevant professional and community networks), and motivation (intention and perceived importance of dissemination) [25]. Results will inform tailoring of training and support strategies. KBs will participate in monthly online meetings (Microsoft Teams) over 6 months. Meetings will include structured check-ins on dissemination activities, discussion of barriers and facilitators, and peer exchange of strategies across provinces. At the first meeting, KBs will determine preferred communication channels for ongoing exchange (eg, WhatsApp; Meta Platforms, Inc, or other group platforms). Meeting minutes and field notes will be documented for process evaluation. In the second meeting, we will provide a workshop on the new gamified version of the Jooay App. The latter meetings will be structured around the COM-B model based on the readiness to disseminate survey results. One meeting will focus on capability (knowledge of Jooay and inclusive leisure resources), another on opportunity (access to relevant professional and community networks), and one on motivation (intention and perceived importance of dissemination). KBs will be responsible for 3 main aspects. The first is identifying adaptive and inclusive leisure activities in their province. The second is sharing updated program information with the Jooay team. The third is disseminating the Jooay App to youth, families, clinicians, educators, and community organizations through existing networks. KBs will document dissemination activities using an online activity log. At the end of the 6-month implementation period, semistructured individual interviews will be conducted with KBs via Microsoft Teams. Interviews will explore dissemination experiences, perceived effectiveness of implementation strategies, contextual barriers and facilitators, and sustainability considerations. Interviews will be audio-recorded, transcribed verbatim, and anonymized. Process data will include monthly meeting notes, KB activity logs, and workshop evaluations. Qualitative data will be analyzed using thematic analysis. Deductive coding will be guided by the CFIR, with attention to intervention characteristics, inner setting, outer setting, characteristics of individuals, and implementation processes. The 2 researchers will independently code transcripts and resolve discrepancies through discussion. Descriptive statistics will summarize readiness-to-implement scores and dissemination indicators.

#### Effectiveness

After signing the consent, during the preparation phase for the pilot study, each participant will receive a tutorial guide for the Jooay App test version. This guide will include instructions on how to navigate and use the app’s various features. Following this, the primary author (EM) will meet individually with each participant, either in person or through an online video call, for an onboarding meeting. During this meeting, participants will be guided through the process of downloading and installing the app on their device, ensuring they feel comfortable with the setup and settings. Participants will have the opportunity to ask any initial questions or concerns regarding the app or study procedures. During the study period, participants will be encouraged to use the Jooay App regularly, engaging with its gamification features to support their participation in leisure activities and work toward their set goals. The research team will be available to answer questions, resolve technical issues, and provide additional guidance as needed. Participants will have direct access to the researcher via email and phone, allowing them to reach out at any time for assistance or clarification. Participants will agree on the periodicity and format of reminders they want to receive to use and engage with the app (eg, weekly, biweekly, monthly email reminders, text messages, or no reminders). The prompts and reminders will be registered in a participant’s “journal” for reference on the types of reminders that might contribute to higher or lower engagement. The preferred frequency and modes will be integrated into the broad public test version as in-app push notifications, email, or text reminders for users. At the end of the study, participants will complete an online survey on Microsoft Form (Multimedia Appendix 1) to capture their overall experiences and perceptions of the Jooay App, covering aspects such as user engagement, satisfaction with specific app features, and any perceived impact on their participation in leisure activities. Participants will be invited to individual semistructured interviews (Multimedia Appendix 2 for interview guide) via Teams to provide clarification and depth to their answers and experiences.

Following the completion of the pilot feasibility study, the gamified version of the app will be deployed to all users at the community level. The date of deployment of the gamified version will serve as the intervention point for an ITS analysis. This design was selected because gamification will be introduced at the app or community level and cannot be randomized across users once it is deployed. The ITS design will allow us to examine whether the introduction of the gamified version is associated with changes in the level and/or trend of engagement outcomes compared with patterns observed during use of the previous nongamified version. Engagement data will be extracted from in-app analytics and aggregated at monthly intervals. The preintervention segment will include monthly data from the previous nongamified version of Jooay, and the postintervention segment will include monthly data after deployment of the gamified version. We will include a minimum of 12 monthly time points before and 12 monthly time points after deployment to allow the estimation of baseline trends, postintervention changes, and seasonal patterns. Seasonality will be assessed through visual inspection of monthly trends. If seasonal patterns are identified, models will include calendar month indicators. We will also document other major app updates, holidays, school calendar periods, or external events that may influence engagement and consider these in adjusted or sensitivity analyses. Engagement outcomes will be measured using both backend and frontend indicators. Backend engagement measures will include app usage frequency, number of accesses, duration of use, and feature usage as captured through in-app analytics. Frontend engagement measures will include engagement with community-based features for youth users and the inclusion of community play and leisure activities within intervention plans developed by clinicians and educators. Additionally, following the deployment of the gamified version, participation in leisure will be assessed using newly co-designed in-app features that capture the number of activities users plan and attend, as well as users’ self-reported satisfaction and perceived achievement. These outcomes will be examined descriptively and longitudinally during the postintervention period to explore patterns of participation over time and their association with app engagement.

### Variables and Outcomes

#### Implementation

The implementation outcomes of this study include both process and intervention-related measures. The implementation process will be analyzed using the CFIR, focusing on outer setting, inner setting, and individual characteristics. The outer setting includes external influences such as patient needs, resources, and organizational networks. KBs recruited from each setting will undergo an initial assessment to identify key considerations, constraints to sustaining the KB role beyond the study, and an intake analysis of individual actions. These factors will be examined in initial KB analyses, monthly KB meetings, and a post hoc multisite analysis. Additionally, external factors such as cosmopolitanism (organizational networking), peer pressure, and policies or incentives supporting leisure-related outcomes will be assessed to determine their role in technology adoption and resource integration. The inner setting includes organizational characteristics affecting implementation, including team culture, intervention compatibility, goal-setting structures, leadership engagement, and implementation climate, which will be analyzed qualitatively through meeting minutes from monthly KB sessions. Individual characteristics, including KBs’ beliefs, knowledge, self-efficacy, and personal attributes influencing implementation, will be assessed during onboarding interviews with the research coordinator. User characteristics (eg, disability type, age, socioeconomic status, language, and technology familiarity) will be analyzed preimplementation during user-centered design activities and pilot testing. These insights will inform KBs’ strategies and interventions.

The implementation and intervention outcomes will assess the expansion of KB networks using social network analysis, as well as the growth in Jooay App users and their engagement levels. Additionally, partners with lived and living experiences (PWLEs) identified increased community awareness of leisure activities as a key implementation outcome, emphasizing its significance in post-COVID public policy and service restructuring. This outcome will be measured through quantitative indicators, including social network analysis and the number of new organizations and activities added to the app during the intervention period, compared with previous registration cycles.

#### Effectiveness

##### Individual Level

###### Feasibility Outcomes

Feasibility will be assessed based on several key indicators: enrollment rate, completeness of data collection, participant retention, and level of technical assistance required. Factors that contribute to the feasibility of using a mobile app in everyday life will also be explored through exit interviews with participants.

###### Engagement

The objective user engagement will be assessed through app analytics data, including interactions with gamification features. This data will provide insights into the frequency of app access as well as engagement with specific gamification elements throughout the study period.

###### Acceptability

The subjective user engagement will be assessed using the System Usability Scale (SUS) [26]. The perceived quality of the Jooay App and impact on the knowledge, attitudes, intentions to participate in leisure activities, and the likelihood of actual change in the target health behavior will be evaluated using a modified version of the Mobile App Rating Scale (MARS) [27].

###### Participation

Participation’s attendance and involvement will be assessed using the “Activity Planner” and “My Summary” features within the Jooay App. The “Activity Planner,” a gamification feature, will allow users to track their planned leisure activities and monitor their completion rates. This feature provided insights into users’ engagement in planned leisure activities over the course of the study. The “My Summary” feature stored users’ responses to self-assessment questionnaires, in which they reflected on their experiences after participating in a specific leisure activity. Users expressed their feelings about the activity by selecting an emoji and identified the benefits gained by choosing relevant domains from the F-word framework for childhood development [28].

### Community Level

The effectiveness of the gamification-based mobile app intervention will be assessed primarily through user engagement metrics collected through app analytics, including the number of clicks, logins, active time spent, access frequency of individual gamification features, comments, and reviews. These data will provide insights into how youth, caregivers, and professionals interact with the app over time. Additionally, differential outcomes will be examined for health care and education professionals, focusing on the integration of community participation into intervention plans and health promotion strategies. For youth, the impact of the intervention will be evaluated based on increased participation in community activities, collected from the “Activity Planner” and “My Summary” features within the Jooay App.

### Analysis

#### Effectiveness

##### Individual Level

Descriptive statistics will be conducted to analyze feasibility outcomes, user engagement with the Jooay App, acceptability, and participation outcomes. Additionally, a hybrid inductive-deductive thematic analysis will be applied to analyze individual interviews to evaluate the app’s impact on user engagement and participation satisfaction.

##### Community Level

Engagement will be measured using a segmented linear regression model to analyze changes over time, including preintervention trends, immediate changes before and after the intervention, and postintervention trends. Where appropriate, models will account for autocorrelation, seasonality, and nonindependence of repeated observations over time. App usage data will capture time spent and interactions with gamification features, with descriptive statistics assessing usage patterns by province, disability, and age among youth participants. Outcomes available only in the gamified version, including planned and attended activities, satisfaction, and perceived achievement, will be analyzed descriptively and longitudinally during the postintervention period rather than included in the primary ITS comparison.

### Implementation Process Evaluation

To assess inner and outer settings, we will use the Child Health Initiatives Limiting Disability-Community Health Inclusion Index (CHILD-CHII) [29]. This tool will identify key characteristics to tailor dissemination strategies, expand KB networks, and enhance user engagement. KBs will also participate in a training workshop and complete the Policymakers’ Information Use Questionnaire (POLIQ; an intention-to-use information tool from the CHILD-BRIGHT (Child Health Initiatives Limiting Disability–Brain Research Improving Growth and Health Trajectories) Knowledge Translation [KT] program, phase I) to assess individual characteristics, including KBs’ beliefs, knowledge, self-efficacy, and personal attributes [30].

### KB Intervention Evaluation

We will conduct a multisite ITS study, accounting for the KB interventions in 5 different provinces. We will then summarize data across several sites (provinces) to evaluate the overall impact of the implementation interventions [31]. Post hoc evaluation will include subgroup analyses for the different types of KBs (health care providers, families, youth, and community organization representatives) and the number of activities listed in each province by population density.

### Patient and Public Involvement Statement

Patients, including youth with disabilities and their families, were involved in the research from the early stages of the project—and will continue to be in this phase of the project. The PWLEs, including youth with disabilities and their families, played a key role in shaping the research questions, ensuring that the study’s focus aligns with their priorities, experiences, and preferences. The development of the research questions was informed by the lived experiences of patients and public stakeholders, particularly regarding the accessibility and impact of leisure activities in the community, a key policy priority for this study. PWLEs codeveloped the user-centered design of the Jooay App, focusing on gamification features, and will help create accessible informational materials for KBs and users. Their feedback also influenced the choice of outcome measures, emphasizing community participation and awareness of leisure activities. PWLEs help inform the choice of dissemination strategies, ensuring that results will be shared meaningfully through social media and networks like Jooay and CHILD-BRIGHT, keeping participants and communities informed throughout the study.

### Ethical Considerations

This study was approved by the McGill University Institutional Ethics Review Board (22-05-070). Participants will provide informed consent, and confidentiality will be maintained throughout the study. For children, parental or caregiver consent will be obtained, and child assent will be sought, using language appropriate to the child’s development. Participants will be informed that their participation is voluntary and that they may withdraw from the study at any time without any negative consequences to their access to services or their use of the Jooay App. As the study involves children and youth with disabilities, app analytics, user behavior data, and participation-related data, additional safeguards will be put in place to ensure privacy and confidentiality. App analytics will be analyzed in aggregate and deidentified form, and personally identifiable information will be removed prior to analysis. Qualitative interview data will be deidentified in transcription and analysis.

All electronic data will be stored on secure, password-protected institutional servers (OneDrive folders) and will be accessible only to authorized members of the research team. Findings will be reported in aggregate form to protect individual users, families, KBs, and regions. Participants will be provided with accommodations during study procedures (eg, interviews) and other research activities. The research team will monitor for participant discomfort or burden, and participants may skip any question or stop participation at any time.

## Results

This study was funded by the CHILD-BRIGHT Network through the Strategy for Patient-Oriented Research, Canadian Institutes of Health Research in September 2025, and data collection is expected to occur from March 2026 to June 2027. As of manuscript submission, 12 KBs have been recruited, the ITS study and data analysis have not yet begun, and the study results for the implementation and effectiveness components are expected to be published in Fall 2027.

## Discussion

### Principal Findings

This study protocol aimed to describe the study that evaluates the effectiveness and implementation of the gamified version of the Jooay mobile app that promotes leisure participation among children and youth with disabilities in Canada.

### Methodological Considerations

One of the key strengths of this study protocol is the use of participatory design with KBs, clinicians, educators, youth, and community partners, ensuring that methods are contextually tailored and inclusive. We have conducted preimplementation strategies, including incorporating human-centered design principles to design a gamification-based mobile app and tailored training for KBs. The gamification-based version of the Jooay App was co-designed in close collaboration with PWLEs and research participants, including youth with disabilities, parents, and health care professionals. The co-design process included individual interviews, co-design group sessions, and usability evaluations. All these strategies can enhance the scalability, sustainability, and accessibility of the Jooay App. Another key strength is the use of mixed methods with ITS design, which can provide a robust framework for evaluating the impact of the Jooay App across diverse provinces and populations. The pilot feasibility study can inform further refinement of the app features before conducting the ITS phase. However, there are several limitations to this study protocol. First, logistical challenges in recruiting KBs and users from diverse backgrounds, along with variability in community partner engagement, may affect representation and reach. Second, the study’s reliance on backend app metrics for engagement data, paired with a small pilot sample size, could limit the generalizability of the mobile app’s effectiveness.

### Dissemination

#### Integrated Knowledge Mobilization Plan—During the Project

We will have frequent meetings (bimonthly) with the Jooay App research team, which includes knowledge users, to give updates on the project’s progress and get their feedback on all steps of project development. Prior to the meetings, the research team members receive a plain-language infographic summarizing the activities, action points, and “questions that need your attention” to be discussed during the meeting. Youth, families, and community partners are offered alternative ways to meet (eg, youth in the past have preferred to meet individually with the principal investigator or research coordinator; parent partners may prefer to have conversations over Facebook; Meta Platforms, Inc or WhatsApp instead of email exchanges). The community at large is informed of project advances through monthly newsletters (opt in through email or social media), social media channels (Instagram; Meta Platforms, Inc, and Twitter), and the Facebook group “Community Connecting to Play.” The KBs will have a monthly meeting to exchange progress, ideas, and advances. Additionally, the progress report will be sent to the CHILD-BRIGHT Steering Committee and the Canadian Institutes of Health Research (the funding agency) annually.

#### Knowledge Mobilization Plan—Outputs of the Project

In collaboration with the team, including PWLEs, educators, clinicians, and others, we will develop a summary of the project’s outcomes in lay language, available in both English and French. The format, including short videos, will be determined in collaboration with our partners. Additionally, we will create an infographic and poster summarizing the outcomes, which will be shared with knowledge users through various social media platforms, including the Jooay Facebook, Instagram, and Twitter accounts, as well as through the CHILD-BRIGHT social media platforms, and our newsletter. Dissemination will be further supported by multiple community partners, including Abilities Online, Ability Center, ParticipACTION, AlterGo, and others, who will share the materials with their members and networks. Additionally, the results of the project will be presented at scientific conferences and will be published in at least 2 scientific journals. In addition, the stakeholder dialogs and knowledge circles will be organized to present results and discuss the scalability and sustainability of the Jooay App and KB networks.

## Supplementary material

10.2196/97008 Multimedia Appendix 1 Jooay app user experience questionnaire.

10.2196/97008 Multimedia Appendix 2 Semistructured interview guide for the Jooay app pilot feasibility study.

## Abbreviations

CFIR: Consolidated Framework for Implementation Research
CHILD-BRIGHT: Child Health Initiatives Limiting Disability–Brain Research Improving Growth and Health Trajectories
CHILD-BRIGHT KT: Child Health Initiatives Limiting Disability–Brain Research Improving Growth and Health Trajectories Knowledge Translation
CHILD-CHII: Child Health Initiatives Limiting Disability-Community Health Inclusion Index
ITS: interrupted time series
KB: knowledge broker
MARS: Mobile App Rating Scale
mHealth: mobile health
POLIQ: Policymakers’ Information Use Questionnaire
PWLEs: partners with lived and living experiences
SUS: System Usability Scale
