# Supplementary material for: Evaluation and Implementation of the Gamified Jooay Mobile Health App: Protocol for a Hybrid Implementation-Effectiveness Study
Source: JMIR Res Protoc. 2026 Sep 4;15:e97008. doi: 10.2196/97008 (PMC13544685; doi:10.2196/97008)
Supplement: Supplementary file 1 [file resprot-v15-e97008-s001.docx]

**Supplemental Material 1**

1. **Please choose your user type ***

- Youth
- Parent/caregiver
- Clinician/educator

**2. Please choose the answer that best describes your experience with the Jooay App.***

| Question | Strongly Disagree | Disagree | Neutral | Agree | Strongly Agree |
| --- | --- | --- | --- | --- | --- |
| I think that I would like to use this app frequently. |  |  |  |  |  |
| I found the app unnecessarily complex. |  |  |  |  |  |
| I thought the app was easy to use. |  |  |  |  |  |
| I think that I would need the support of a technical person to be able to use this app. |  |  |  |  |  |
| I found the various functions in this app were well integrated. |  |  |  |  |  |
| I thought there was too much inconsistency in this app. |  |  |  |  |  |
| I would imagine that most people would learn to use this app very quickly. |  |  |  |  |  |
| I found the app very challenging to use. |  |  |  |  |  |
| I felt very confident using the app. |  |  |  |  |  |
| I needed to learn a lot of things before I could get going with this app. |  |  |  |  |  |

**3. Please choose the answer that best describes your experience with the Jooay App.***

| Question | Strongly Disagree | Disagree | Neutral | Agree | Strongly Agree | Not Applicable |
| --- | --- | --- | --- | --- | --- | --- |
| Jooay helped me find new leisure activities to do near me/The Jooay app helped me learn more about leisure activities for my child in our community/Using the Jooay app increased my knowledge of local leisure activities I can share with clients. |  |  |  |  |  |  |
| Using the Jooay app made me want to try new leisure activities/The Jooay app encouraged me to support my child in trying new leisure activities/The Jooay app motivated me to recommend or support leisure participation among my clients. |  |  |  |  |  |  |
| Jooay made it easier and more fun for me to plan my leisure activities/I found the Jooay app useful in helping me find and plan leisure activities for my child/I found the Jooay app to be a helpful tool in planning or discussing leisure with clients and families. |  |  |  |  |  |  |
| Since using the Jooay app, I have tried new leisure activities or gone to new places for fun/Since using Jooay app, my child has tried new leisure activities. |  |  |  |  |  |  |
| Jooay felt right for me and matched the kind of things I like to do/The Jooay app was appropriate and relevant to my child's needs and interests/The Jooay app fits well with how I support families in daily practice. |  |  |  |  |  |  |
| I want to keep using the Jooay app in the future/I would continue to use the Jooay app with my child./I would continue to use the Jooay app in my clinical work. |  |  |  |  |  |  |
| I would tell my colleagues to try the Jooay app too/I would recommend the Jooay app to other parents/I would recommend the Jooay app to other clinicians or families. |  |  |  |  |  |  |
| I think that I am pretty good when I use this app/I am satisfied with my performance when I use this app to support my child’s participation/I feel like a competent person when I use this app to support my clients. |  |  |  |  |  |  |
| I feel free to decide what activities to do on this app/In this app, I have different options to choose from for my child/I feel free to use this app in a way that works for my practice. |  |  |  |  |  |  |
| I feel supported by others when using this app/I feel that I am understood by others who use this app (e.g., families, colleagues). |  |  |  |  |  |  |

## Additional Questions

4. What is your overall star rating of the app? * ⭐⭐⭐⭐⭐

5. Do you have anything else that you want to tell us?
