# Supplementary material for: Evaluation and Implementation of the Gamified Jooay Mobile Health App: Protocol for a Hybrid Implementation-Effectiveness Study
Source: JMIR Res Protoc. 2026 Sep 4;15:e97008. doi: 10.2196/97008 (PMC13544685; doi:10.2196/97008)
Supplement: Supplementary file 2 [file resprot-v15-e97008-s002.docx]

**Supplementary Material 2**: Semi-Structured Interview Guide for the Jooay App Pilot Feasibility Study

**Purpose**
This semi-structured interview guide was used in the *Jooay App Pilot Feasibility Study* to explore participants’ experiences, engagement, and perceived usefulness of the app. The guide was adapted for youth, parents/caregivers, and clinicians, allowing flexibility to probe based on participants’ responses. Each interview lasted approximately 45–50 minutes.

### **Section 1: Introduction**

- Thank the participants for their time and participation.
- Explain the purpose of the interview: to learn about their experiences using the Jooay app and to understand what worked well, what could be improved, and how it may support participation in leisure activities.
- Confirm consent to record the session for transcription and analysis.

### **Section 2: General Experience**

1. Can you describe your overall experience using the Jooay app?
   - (Probe: What were your first impressions?)
2. How did you typically use the app? (e.g., daily, weekly, occasionally)
3. What motivated you to try the app?
4. How do you typically find or plan leisure activities?

### **Section 3: Usability and Functionality**

1. What did you think about the app’s design and layout?
2. Were there any features you found especially helpful or frustrating?
3. How easy or difficult was it to navigate the app?
4. Did you experience any technical issues or bugs? If yes, please describe them.
5. Were there any parts of the app that were confusing or unclear?

### **Section 4: Gamification Features**

1. How did you feel about the gamification features (e.g., earning gems, using the avatar, completing “My Plan”)?
2. Did these features make the app more enjoyable or motivating for you? Why or why not?
3. Which gamification feature did you like the most?
4. Were there any features you did not like or found unnecessary?

### **Section 5: Engagement and Motivation**

1. What motivated you to continue using (or stop using) the Jooay app?
2. Would you consider continuing to use the app after the study? Why or why not?
3. Did using the app make it easier for you to plan or participate in leisure activities?
4. Did the app encourage you to try new activities or explore new places?

### **Section 6: Accessibility and Inclusivity**

1. Did you find the app accessible and inclusive for your needs (or your child’s needs)?
2. Were there any accessibility challenges (e.g., language, visual design, readability, or functionality)?
3. Do you have suggestions to make the app more inclusive for children and youth with different needs?

### **Section 7: Social Interaction**

1. How did you find the communication or interaction features within the app (if applicable)?
2. Would you have liked more opportunities to connect with others through the app? If yes, how?

### **Section 8: Overall Perceptions and Recommendations**

1. What did you like most about the Jooay app?
2. What would you change or improve?
3. How likely are you to recommend the Jooay app to others (e.g., peers, families, or colleagues)?
4. Is there anything else you would like to share about your experience?

**Note:**
This guide was developed and refined collaboratively by the Jooay research team. The interviewer was trained in qualitative interviewing and followed a conversational, flexible approach to allow participants to elaborate on their experiences.
